# The molecular conformation, but not disaggregation, of humic acid in water solution plays a crucial role in promoting plant development in the natural environment

**DOI:** 10.3389/fpls.2023.1180688

**Published:** 2023-05-03

**Authors:** Javier Aranaz, David de Hita, Maite Olaetxea, Oscar Urrutia, Marta Fuentes, Roberto Baigorri, Maria Garnica, Maria Movila, Angel M. Zamarreño, Javier Erro, Enrique Baquero, Gustavo Gonzalez-Gaitano, Jose Ignacio Alvarez, Jose M. Garcia-Mina

**Affiliations:** ^1^ Institute for Biodiversity and Environment BIOMA, University of Navarra, Pamplona, Spain; ^2^ Department of Chemistry, Faculty of Sciences, University of Navarra, Pamplona, Spain

**Keywords:** humic acids, supramolecular, plant growth, metal complexation, soil organic matter, dissolved organic matter (DOM)

## Abstract

Many studies have shown the capacity of soil humic substances (HS) to improve plant growth in natural ecosystems. This effect involves the activation of different processes within the plant at different coordinated molecular, biochemical, and physiological levels. However, the first event triggered by plant root-HS interaction remains unclear. Some studies suggest the hypothesis that the interaction of HS with root exudates involves relevant modification of the molecular conformation of humic self-assembled aggregates, including disaggregation, which might be directly involved in the activation of root responses. To investigate this hypothesis, we have prepared two humic acids. A natural humic acid (HA) and a transformed humic acid obtained from the treatment of HA with fungal laccase (HA enz). We have tested the capacity of the two humic acids to affect plant growth (cucumber and Arabidopsis) and complex Cu. Laccase-treatment did not change the molecular size but increased hydrophobicity, molecular compactness and stability, and rigidity of HA enz. Laccase-treatment avoided the ability of HA to promote shoot- and root-growth in cucumber and Arabidopsis. However, it does not modify Cu complexation features. There is no molecular disaggregation upon the interaction of HA and HA enz with plant roots. The results indicate that the interaction with plant roots induced in both HA and laccase-treated HA (HA enz), changes in their structural features that showed higher compactness and rigidity. These events might result from the interaction of HA and HA enz with specific root exudates that can promote intermolecular crosslinking. In summary, the results indicate that the weakly bond stabilized aggregated conformation (supramolecular-like) of HA plays a crucial role in its ability to promote root and shoot growth. The results also indicate the presence of two main types of HS in the rhizosphere corresponding to those non-interacting with plant roots (forming aggregated molecular assemblies) and those produced after interacting with plant root exudates (forming stable macromolecules).

## 1 Introduction

Many studies have demonstrated the functional links between soil fertility, soil organic matter (SOM), and the fraction of organic matter in soil solution (dissolved organic matter, DOM) (Chen et al., 2004; Magdoff and Weil, 2004). This fact has been related to the ability of specific components of SOM and DOM to affect plant development by improving plant mineral nutrition and root functionality (Chen et al., 2004; Olaetxea et al., 2018). Among these components, the organic fraction extracted from the soil matrix with alkali, denoted humic substances (HS), has particular relevance (Chen et al., 2004; Olaetxea et al., 2018). Many studies have shown the ability of HS to increase the pool of plant-available nutrients due to their capacity to form stable complexes with several micronutrients (Olaetxea et al., 2018). Likewise, other studies have reported the ability of HS to interact with plant roots inducing relevant changes in plant metabolism at molecular, enzymatic, and physiological levels (Trevisan et al., 2010; Olaetxea et al., 2018 and references there in). However, the primary event occurring in cell membranes at the root surface linked to HS interaction that triggers the subsequent chain of biochemical events remains unknown.

It has been accepted for many years that humic substances tend to form self-assembled aggregates in water solution depending on some experimental variables such as pH, ionic strength, or the presence of polyvalent cations, mainly metals (Stevenson, 1994). In some cases these humic super-aggregates have been considered supramolecules (Piccolo, 2002; Baigorri et al., 2007; Karpiouk et al., 2012; Chaaban et al., 2021; Gerzabek et al., 2022). Supramolecular chemistry involves concepts such as inter-molecular self-assembly and non-covalent attractive forces but are included in specific frameworks involving order, design, molecular recognition, emerging new properties, and functions; synergy versus additive (Menger, 2002; Lehn, 2005). Self-assembled supramolecules usually have new properties that do not have the individual components of the supramolecule at least at the same level. To what extent do the humic molecular aggregates meet these conditions to be considered supramolecules? It becomes clear that specific research is needed to clarify this open question. For this reason, we use in the text terms similar to “molecular self-assembled aggregates” or “supramolecular-like” when referring to the configuration of humic systems.

Some studies have reported that HS interaction with root cells affects root hydraulic conductivity and aquaporin activity through signaling pathways dependent on ABA (Olaetxea et al., 2015; Olaetxea et al., 2019). These effects of HS are directly linked to their distinct molecular conformation in solution (Olaetxea et al., 2021). Likewise, other studies have reported that high concentrations of HS cause the fouling of pores at the root surface, inhibiting water uptake and inducing drought stress (Asli and Neumann, 2010). In this sense, some studies proposed that the beneficial action of HS derives from an HS-mediated mild and transient stress that has a priming effect enhancing the plant’s capacity to grow under normal and stressful conditions (Olaetxea et al, 2015; Olaetxea et al., 2018; Souza et al., 2022). This fact has been observed in some studies (De Castro et al., 2021). In this framework, it has been proposed that one crucial step of this process could be the HS conformational changes (or even the HS molecular disaggregation) induced by their interaction with some types of root exudates, such as polycarboxylic acids, in the rhizosphere (Piccolo et al., 1996; Canellas et al., 2008; Olaetxea et al., 2018). If this hypothesis is true, it becomes clear that the supramolecular-like conformation of HS (Piccolo, 2002; Baigorri et al., 2007), reflected in the presence of molecular aggregates stabilized by weak intermolecular forces, would play a relevant role in the whole process.

In order to investigate this hypothesis, we should study the relevance of the self-assembled aggregate conformation of a model humic acid in the evolvement of some aspects of its biological and chemical activities. For this, we need to reduce the inter-molecular aggregation (supramolecular-like conformation) of a model humic acid by increasing its macromolecular character and evaluating the effects of these changes in its biological and chemical activities.

Some studies have shown that the controlled catalytic oxidation of different HS and other biomolecules leads to the transformation of weak inter-molecular interactions into covalent chemical bonds that confers high molecular rigidity and structural stability (Piccolo et al., 2005). These treatments induce inter and intra- molecular crosslinking, leading to a loss in the biomolecule’s molecular self-assembly character and increasing its macromolecular character. Among these treatments, fungal laccase enzymes have proven to be very efficient (Cha et al., 2017).

In this context, we have subjected a model humic acid (denoted HA) to laccase-mediated catalytic mild oxidation to obtain an HA analog with higher macromolecular character and less molecular-assembly features (denoted HA enz) and its capacity to both stimulate plant growth, and complexing metals has been compared to that of HA.

The biological activity of HA and HAenz was evaluated by measuring their ability to affect shoot and root growth and root morphology in cucumber and Arabidopsis plants, while one aspect of their chemical activity was studied by measuring the complexation of Cu in solution. HA size distribution changes in nutrient solution in the presence of plant roots were determined by dynamic light scattering (DLS), and the conformational and structural changes were evaluated using potentiometric analysis, FTIR, 1H and 13C NMR, thermogravimetric analysis, hydrophilicity (wettability and contact angle), and synchronous fluorescence.

## 2 Materials and methods

### 2.1 Extraction and purification of a leonardite humic acid (HA)

The specific methodology for humic acid purification and characterization was extensively described in previous studies (Garcia-Mina et al., 2004; Aguirre et al., 2009; Mora et al., 2010). Briefly, 5 g of non-dried leonardite was extracted with 0.1 M NaOH under N_2_ atmosphere. After 24 h stirring at 25 °C in darkness, the supernatant containing the whole humic extract was separated from the solid fraction by centrifugation at 7650 × g for 30 min. The supernatant containing the whole humic extract was acidified to pH 1.5 with concentrated HCl. After 24 h, the precipitate containing the humic acid (HA) was separated by centrifugation (4500 g for 30 min) and freeze-dried. HA sample was obtained from an open mine of Leonardite (Danube basin).

### 2.2 Synthesis of an HA analog through laccase treatment (HA enz)

The HA analog was obtained through the treatment of HA with fungal laccase, as described below.

Cha et al. (2017) described the complete methodology of the reaction procedure. Briefly, 5g of HA were dissolved in 1L of (75% of a buffer dissolution and 25% of ethanol). 0.1g of laccase enzyme was added. It was stirred for 24 hours at 25rpm and 36°C. Once the reaction was finished, an amount of HCl was added until pH turned 1-2. The solution was centrifuged, and the precipitate was freeze-dried before its characterization and application in plant experiments.

### 2.3 Physicochemical characterization of HA and HA enz

#### 2.3.1 Elemental analysis

The elemental analysis of the samples (the percentage of C, H and N; O was calculated by difference) was made using a Thermoscientific FlashSmart CHNS.

#### 2.3.2 Potentiometric titrations

Potentiometric titrations were carried out using a Metrohm Titrando 809 instrument under N_2_ atmosphere as described in Garcia-Mina (2007). A combined pH glass electrode of the same company registered the pH.

Before starting the titration procedure, an H+-cationic exchange resin (Amberlite IRA-118H^+^) was added to lower the pH of the initial humic solution to a pH of 3.5 and avoid precipitations. The titration studies were performed in samples at a concentration of 20 mg of C of the molecular system in 0.5mL of 0.1M NaOH, 5mL of NaClO_4_, and deionized water until the final volume was 45mL. The ionic strength (I) was fixed at 0.1M adding KNO_3_ and the initial pH of the samples resulted in 2.9. Samples were then titrated with the sequential addition of 0.05mL of 0.1M NaOH, and the titration procedure finished when pH values remained invariable (± 0.01) for 5 minutes.

#### 2.3.3 FTIR spectra

Functional group distribution of HA and HA enz was characterized using ATR-FTIR spectroscopy Attenuated Total Reflectance (ATR). Infrared spectra were recorded over the 4000-600 cm^-1^ range with a resolution of 4 cm^-1^ in a Shimadzu IRAffinity-1-S.

#### 2.3.4 HPSEC

Molecular size distributions were assessed using a chromatographic system consisting of a Waters 2795 Alliance followed by a Waters 2996 Photodiode Array Detector set at 280 nm. Size exclusion separation occurred through a XBridge BEH 200 Å SEC 3.5 µm 7.8x150 mm column (Waters).

#### 2.3.5 ^1^H-liquid NMR

1H NMR spectra were recorded in dissolved samples by using a Varian Unity-300 instrument. Frequency was fixed at 300MHz, with 90° pulse angle, a sweep width of 4000 Hz, and a line broadening of 0.5Hz using a 5mm multinuclear probe. Sodium 3-trimethylsilyl-propionate-2,2,3,3,-d4 (TSP) was added to the samples to provide a chemical shift standard.

#### 2.3.6 ^13^C NMR

Freeze-dried samples were measured in a Varian Unity 300 spectrometer. Cross-polarization magic angle spinning technique was applied (CPMAS) with a spinning speed of 5kHz, a frequency of 75.429 MHz, 90° pulse width, 69ms acquisition time, and 1.0s delay.

#### 2.3.7 Synchronous fluorescence spectroscopy

Fluorescence spectra were performed on a Shimadzu RF-6000 cuvette fluorescence spectrophotometer. Solutions of 10 mg L^-1^ of organic carbon were prepared in 0.05M of NaHCO_3_. Synchronous spectra were collected between 380nm and 690nm and in the same conditions as described in Peuravuori et al, (2002).

#### 2.3.8 UV-visible spectroscopy

UV–visible spectroscopy Spectroscopic analyses of the samples were performed on a HP8543 spectrophotometer with a 1 cm quartz window cuvette. The absorbance at 415nm and the E400/E600 ratio were measured (Fuentes et al., 2006).

#### 2.3.9 Dynamic light scattering (DLS) study

Size distributions were obtained by DLS at a scattering angle of 90° using a DynaPro-MS/X photon correlation spectrometer equipped with a 248 channel multi-tau correlator and a Peltier effect thermostatization unit. The size distributions were obtained from the intensity autocorrelation function by regularization analysis with the implemented DynamicsTM software, and the hydrodynamic radii (Rh) deduced from the diffusion coefficients through the Stokes-Einstein equation. All the experiments were carried out at 25°C.

#### 2.3.10 Thermal study

The thermal degradation of the samples was studied with a SDT650 (TA Instruments) equipment that recorded both the continuous mass variations (TG, thermogravimetric curve) and the heat flux signal (DSC, differential scanning calorimetry signal). The equipment was pre-calibrated with weight standards for the TG signal and with calibration sapphire for the DSC signal. About 10 mg of both compounds, HA enz and HA, were placed in 90 µL alumina crucibles previously tared. The samples were subjected to a heating program that included a stabilization step at 35°C and a heating ramp from 35° to 1100°C, at a rate of 5°C/min. The balance maintained a constant N_2_ purge at 100 mL/min.

Tests were carried out with samples under two different conditions during heating: one, to study degradation in an inert atmosphere (pyrolysis) with a flow of 50 mL/min of N_2_ over the sample; the other, to study degradation in an oxidizing atmosphere (combustion) with a flow of 50 mL/min of pure O_2_.

The interpretation of experimental curves and weight loss percentages, evaluation of areas under the curve, and integration for thermodynamic calculations were carried out using TRIOS software from TA Instruments.

#### 2.3.11 Hydrophilicity, wettability and contact angle (WCA)

The powdered humic acid samples were pressed to obtain pellets for contact angle measurement. The sessile drop method was used to determine the static contact angle. This method involves placing a drop of water with a syringe on the sample’s surface. The contact angle was measured by adjusting the shape of the drop to an ellipse. The video-measuring system used was OCA15 EC equipment (Dataphysics Instruments), and the software was SCA20 from the same company, while a 5 mL Hamilton syringe was used to add the water droplets.

The ARCA (Advancing and receding contact angle) method was used to measure the dynamic contact angle. A 5 μL distilled water droplet was placed on the substrate surface, but in this case, the syringe was continuously introduced into the droplet. According to the ARCA procedure of the SCA20 software, the dynamic contact angles, forward and reverse, were measured, by making the drop grow and shrink 2.5 μL, at a rate of 0.1 μL.s-1 for five cycles, with a time of 3 seconds between cycles. The advancing and receding angles are the maximum and minimum angles measured when the droplet contact line on the surface advances and recedes. The repellency was measured from the hysteresis (difference between advancing and receding angles). The lower the hysteresis value, the lower the force required to move a water droplet on a surface and, thus, the higher the hydrorepellency of the material.

### 2.4 Plant material, culture conditions and treatments

#### 2.4.1 Experiment 1. Effects of HA and HA enz on the growth of cucumber plants, and measurement of their size-distribution in solution by DLS

The process of germination of cucumber seeds (Cucumis sativus L. cv “Ashley”) was carried out on perlite and moistened filter paper (1mM CaSO_4_), in darkness, and a germination chamber. The seeds were first introduced in a 1mM CaSO_4_ solution for one hour with stirring. One week after germination, plants were transferred to 0.8L receptacles in a hydroponic solution. The nutrient solution was composed of the following concentration of salts: 0.63 mM K_2_SO_4_, 0.5 mM KH_2_PO_4_, 0.5 mM CaSO_4_, 0.30 mM MgSO_4_, 0.25 mM KNO_3_, 0.05 mM KCl, 0.87 mM Mg(NO_3_)_2_, 40 μM H_3_BO_3_, 4 μM MnSO_4_, 2 μM CuSO_4_, 4 μM ZnSO_4_, and 1.4 μM Na_2_MoO_4_, 5 mg Kg-1 of iron as Fe‐EDDHA chelate (80% ortho‐ortho isomer). The pH of the nutrient solution was held at 6. The growth chamber conditions were fixed at 25°C day/21°C night, 65-70% humidity, and with 16/8 hours day/night photoperiod (irradiance: 175 μmol/m^2^ s^‐1^). Plants were grown for 7 days. At this time point of the experiment, the different treatments were applied. After 72 h from the treatment, the plants were harvested. Each treatment has five replicates of one plant per replicate. The plant material (shoot and roots separately) was dried at 60 °C for 48 h and was used to obtain fresh and dry matter production. Two treatments were applied: HA and HA enz, at a concentration of 200 mgL^-1^ of organic carbon.

In order to determine the Rh of HA and HA enz in the nutrient solution in the presence of plants and without plants, samples of the nutrient solution for the treatments and a nutrient solution with the treatments but without plants were collected for 1, 4, 24, 48 and 72 hours after treatments. The samples were filtered through 0.45 (for all harvest times) or 0.1 µm (for 1, 4, 24 h) filters before DLS measurements.

#### 2.4.2 Experiment 2. Effect on the size distribution of HA-Fe(III) complexes of the interaction with cucumber plant roots

Cucumber plants were prepared as described in Experiment 1. Two culture conditions were compared. Control plants receiving the nutrient solution and Fe (III) as Fe-EDDHA, and a treatment receiving the nutrient solution but Fe (III) as HA-Fe(III) complex. Each treatment has five replicates of one plant per replicate. Plants were harvested 72h from the onset of treatments, and Fe concentration in roots and leaves was determined of dry material using ICP-OES previous microwave-assisted acid digestion of the samples. Samples of the nutrient solution of the treatments were collected after 1 and 4 h from the onset of treatments. The variation of the Rh of HA-Fe(III) in nutrient solution was evaluated by DLS.

#### 2.4.3 Experiment 3. Effects of HA and HA enz on the growth and root morphology of Arabidopsis plants

Arabidopsis (Arabidopsis thaliana) plants used in this study were of the Col-0 ecotype. The seeds were sterilized with diluted bleach for one minute. Seeds were sowed in agarose gel. The receptacles were filled with water during the first 10 days of the germination process. Afterward, the growing process continued in a nutrient solution. The nutrient solution was composed of the following concentration of salts: MgSO_4_·7H_2_O 92.43g/L, KH_2_PO_4_ 34.02g/L, KNO_3_ 63.19g/L, Ca(NO_3_)_2_·4H_2_O 177.11g/L, Fe EDDHA 12.4g/L, KCl 3.73g/L, MnSO_4_·H_2_O 1.69g/L, CuSO_4_·5H_2_O 0.37g/L, ZnSO_4_·7H_2_O 0.58g/L, H_3_BO_3_ 3.09g/L, (NH_4_)_6_Mo_7_O_24_ 0.09g/L. The pH of the nutrient solution was held at 6. The growth chamber conditions were fixed at 21°C day/18°C night, 75% humidity, and with 10/14 hours day/night photoperiod (irradiance: 175 μmol/m^2^ s^‐1^). Plants were grown for 15 days. After the growing process, the treatments were applied. Each treatment has five replicates of five plants per replicate. After 7 days, the plants were harvested. As in the case of cucumber, the plant material (shoot and roots separately) was dried at 60 °C for 48 h and was used to obtain fresh and dry matter production. Two treatments were applied: HA alone and HA enz, at a concentration of 20 mgL^-1^ of organic carbon.

Specific segments of fresh plant roots were studied using optical microscopy to evaluate lateral root production. Root photographs were performed using an Olympus BX51-TF microscope with a multi-viewing system.

The accumulation of the number of tips for each of the samples was quantified though a methodology of image analysis. Also, the root morphology of the samples was studied applying WinRHIZO image analysis system to five entire scanned roots for each of the treatments (n=5). WinRHIZO performs a morphological (length, area, volume etc.), topological, architectural and color analysis of the image.

### 2.5 Cu complexation by HA and HA enz

The Cu(II) complexing capacities of HAn and HA enz as well as the stability constants of the Cu(II)-HA complexes were determined by the quenching of fluorescence according to the method proposed by Ryan and Weber (1982) and the methodology detailed in Plaza et al. (2005) and Fuentes et al. (2013). Briefly, stock solutions of 30 mg HA or HA enz L-1 were prepared in 0.1M KNO_3_ and the pH was adjusted to 8. Aliquots of the stock solutions were diluted with equal volumes of solutions of different concentrations of Cu(NO_3_)_2_ in 0.1M KNO3 at pH 8, so that the final solutions had 15 mg L^-1^ HA or HA enz, 0.1M KNO_3_ and 0, 7.5, 15, 25, 40 or 60 µM Cu(II). Solutions were left overnight at room temperature before fluorescence measurements. Fluorescence measurements were performed on a Shimadzu RF-6000 Spectrofluorophotometer.

### 2.6 Statistical analysis

Significant differences between means of treatments were calculated using the LSD Fisher test.

## 3 Results and discussion

### 3.1 Laccase treatment induced significant changes in the physicochemical features of HA

**Figure 1:**
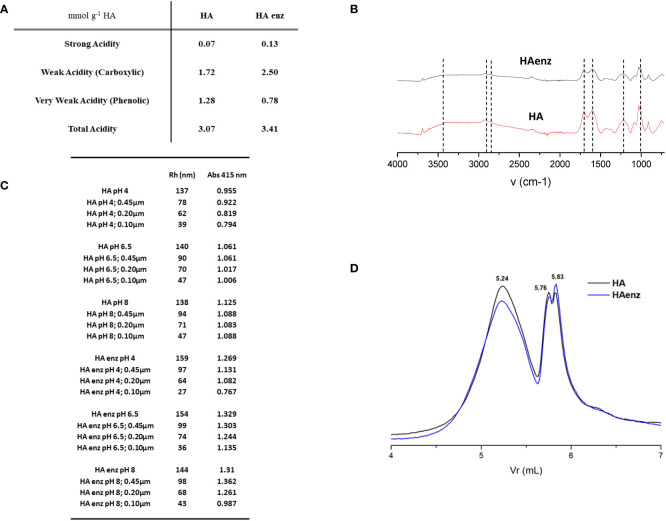
Physico-chemical features of HA and HA enz. **(A)** Potentiometric analysis; **(B)** FTIR; **(C)** Molecular size distribution by DLS. Hydrodynamic Radius (Rh) values in nm; **(D)** Molecular size distribution by HPSEC.

In order to check if the supramolecular-like structure of HA plays a role in the evolvement of some aspects of its biological and chemical activities, we treated HA with laccase enzyme to promote intramolecular crosslinking in HA thus decreasing its molecular-aggregation and increasing its macromolecular character. In line with this objective, the titration of HA and HA enz samples showed that the treatment with laccase produced significant changes in the concentration of those groups with weak acidity, usually associated with carboxylic groups and very weak acidity linked to phenolic groups (**Figure 1A**). Thus, carboxylic groups increased by around 45%, while phenolic groups decreased by around 30%. This result is in line with other studies (Cha et al., 2017), and with the mechanism of action of the laccase enzyme that involves the single-electron oxidation of phenolic groups forming phenoxyl radicals that can couple with adjacent structural moieties or evolve into more oxidized groups (Cha et al., 2017). It is important to consider that besides the conformational changes, laccase treatment also causes changes in the distribution of acidic functional groups. This fact is relevant to interpret the results obtained from the study concerning biological and chemical activities since the relative amount of phenolic and carboxylic groups might influence these parameters. In this sense, however, previous studies showed that the ability of HA to affect root growth and morphology is more related to whole structural properties (like hydrophobicity: hydrophilicity ratio or lability: recalcitrance ratio) than to the relative proportion of carboxyl and phenolic groups (García et al., 2016).

Likewise, the elemental composition of HA (42.12 ± 1.84% C; 3.04 ± 0.27% H; 53.68 ± 2.08% O; 0.97 ± 0.06% N) and HA enz (47.33 ± 0.78% C; 3.47 ± 0.12% H; 48.10 ± 0.89% O; 1.10 ± 0.08% N) indicates that the oxidation process is associated with a oxidation-degradation of O-containing groups that might be related to some type of carbohydrates, or small highly oxidized molecules.

FTIR spectra of HA and HA enz presented similar profiles: the bands corresponding to the O-H deformation of aliphatic O-H and C-O deformation of polysaccharides (1000-1100 cm^-1^). The bands attributed to C=O stretching vibration 1715 cm^-1^ correspond to COOH, ketones, aldehydes, and esters, and the C-O stretching and OH deformation in COOH (1283 cm^-1^). The aliphatic C-H stretching at 2925-2850 cm^-1^. The aliphatic C-H (1431 cm^-1^) and the OH deformation and C-O stretching in phenols (1381 cm^-1^), as well as the aromatic C=C at 1620 cm^-1^ (**Figure 1B**). (Stevenson, 1994; Fuentes et al., 2007).

It is expected that the molecular crosslinking induced by laccase treatment leads to more compacted structures that may affect the molecular size distribution in the solution (Piccolo et al., 2005). Some studies described an increase in the size of humic acids resulting from oxidative polymerization (Piccolo et al., 2005). Likewise, treating lignosulfonates with laccase led to significant changes in molecular weight (Areskogh et al., 2010). In our case, however, the results obtained from HPSEC showed a slight decrease in the peak corresponding to larger sizes and a corresponding increase in that linked to smaller sizes after laccase treatment (**Figure 1D**). However, the differences were minimal (**Figure 1D**). These results were also reflected in the DLS study investigating the influence of pH and pre-filtration treatment on HA- and HA enz- molecular size (**Figure 1C**; **Figure S1**). Considering the pH of 6.5 and the sample filtered with 0.1 µm, we observe that HA presented an Rh value of 46 nm while HA enz has a value of 36 nm (**Figure 1C**).

On the other hand, sample filtration was associated with apparent changes in the Rh that decreased depending on the filter used (**Figure 1C**). Only at pH 4 were these changes associated with a concomitant decrease in the Abs at 415 nm, indicating a decrease in the HA concentration resulting from sample filtration. At pH 6.5 and 8, there was no decrease in Abs 415, thus indicating the strong influence of those fractions with a larger size on the average Rh measured by DLS, although their relative concentration in the system is very low (**Figure 1C**). These results show a good concordance between molecular size variation determined by DLS and HPSEC.

**Figure 2:**
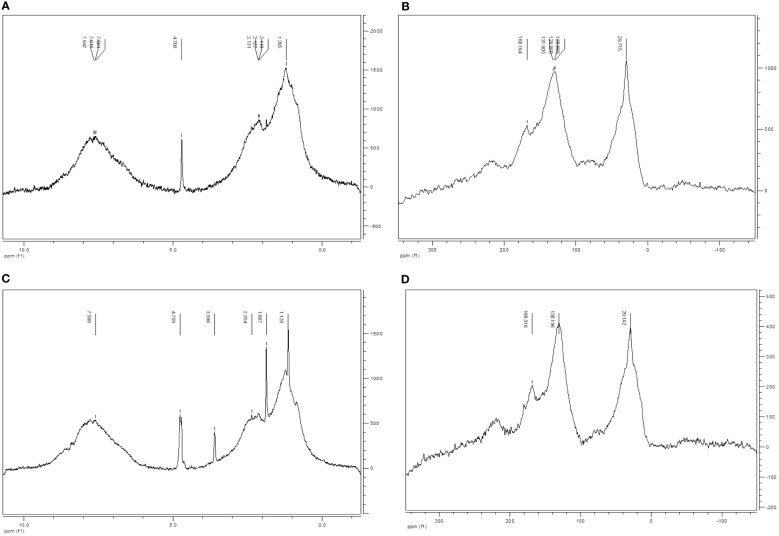
Characterization of HA and HA enz by liquid-state 1H NMR and solid-state 13C NMR. **(A)** 1HT2 NMR of HA; **(B)** 13C CP-MAS NMR of HA; **(C)** 1HT2 NMR of HA enz; **(D)** 13 C CP-MAS NMR of HA enz; Proton-type distribution (0.5-3 ppm (Alkyl, alcohol, RNH2); 3-4.5 ppm (phenol, ArNH2); 4.5-8.5 ppm (phenol, aromatic)) (Carbon-type distribution (0-45 ppm (unsubstituted aliphatic C); 45-60 ppm (N-alkyl and methoxyl C); 60-95 ppm (aliphatic C-O); 95-110 ppm (aliphatic O-C-O); 110-140 ppm (aromatic C); 140-160 ppm (O-aryl); 160-185 ppm (Carboxyl C).

^1^H- liquid NMR and ^13^C- solid NMR did not show apparent differences between the main primary structures of HA and HA enz since the two humic acids presented qualitatively almost the same C and H distribution (**Figures 2A–D**). There are two facts that can be underlined. The amount of carbohydrates in HA is very low probably due to the biotic and abiotic degradation associated with the humification process (Stevenson, 1994). This faction is even lower in laccase treated-HA probably due to an additional oxidation linked to the treatment. This conclusion is in line with the results obtained from the elemental analysis. On the other hand, the aromatic character of HA enz (45.1% of C) is higher than that of HA (40.4%). This increase in the aromatic character might be associated with an increase in hydrophobicity.

This fact might be associated with increased molecular compactness and rigidity from the oxidative polymerization observed in several studies (Piccolo et al., 2005).

**Figure 3:**
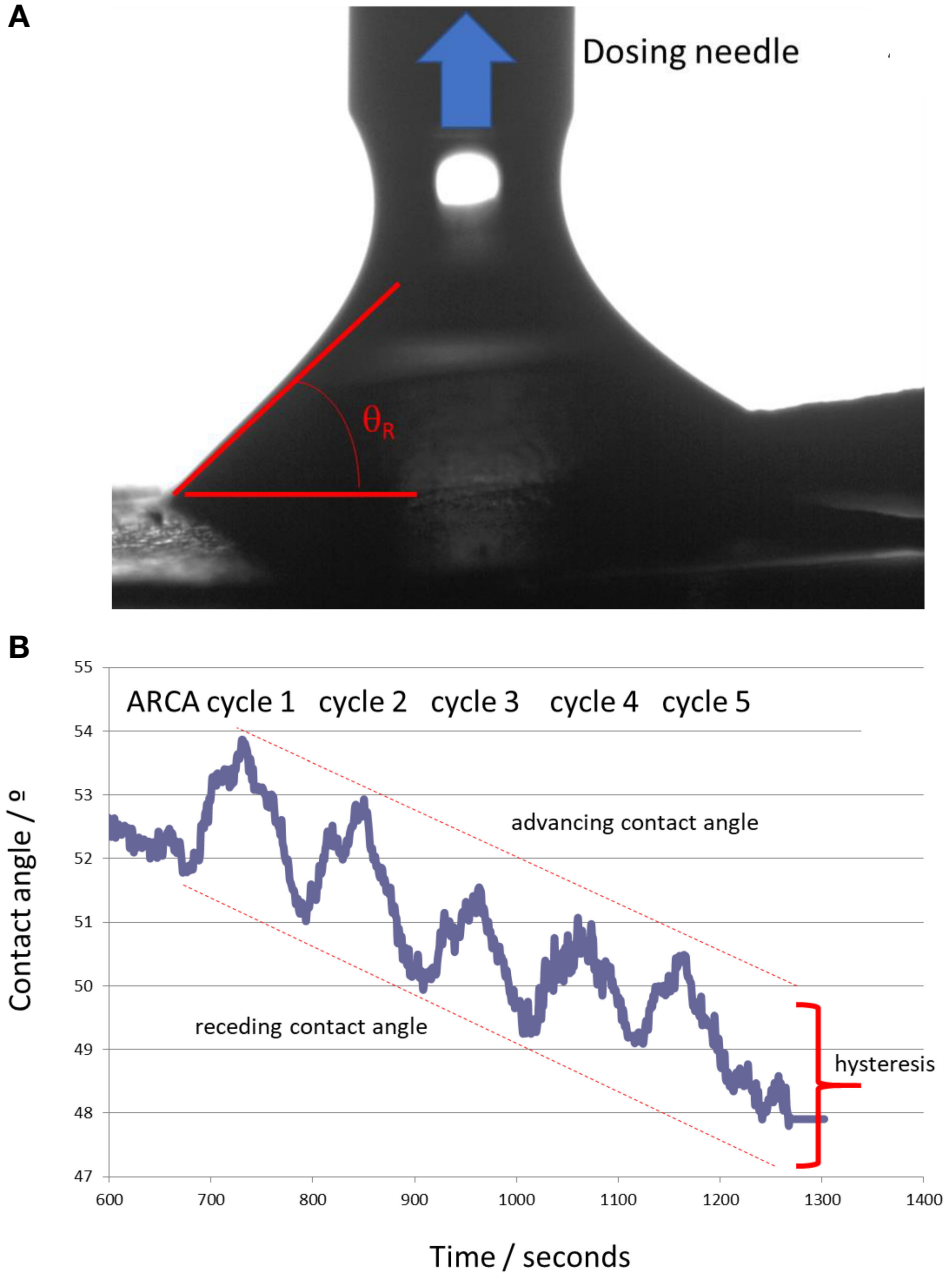
Dynamic contact angle study: **(A)** determination of the receding contact angle during ARCA experiments; **(B)** Results of the five cycles of the ARCA procedure. The maximum values were considered as the advancing contact angles, whereas the minimum values of each cycle were the receding contact angles. The hysteresis value, related to the wettability of the sample, was calculated as the average difference of the five cycles of the experiment.

This potential increase in hydrophobicity resulting from laccase treatment indicated by ^13^C-NMR may be confirmed by measuring the hydrophilicity, wettability, and contact angle (WCA) of powdered humic samples (**Figure 3**). The results showed that HA presented a very high hydrophilicity being the sessile drop rapidly absorbed by the material. However, the HA enz presented a static contact angle of 57.22° that showed significant hydrophilicity but was lower than that of HA (**Figure 3**). The ARCA method confirmed this result by measuring the surface’s wettability through the dynamic contact angle (**Figure 3**). The difference between the advancing and receding contact angles (hysteresis) is mainly related to the work of adhesion, that is, the work that should be applied to separate two phases in contact with each other. Whereas HA was rapidly dissolved, HA enz presented a hysteresis value of around 2.40°, which is very low and indicates a repellence of water by HA enz (**Figure 3**). Consequently, these results confirm that HA enz is more hydrophobic than HA.

As mentioned above, ^13^C-NMRstudy and WCA suggest that laccase treatment is associated with an increase in molecular hydrophobicity that might be related to a concomitant increase in both molecular compactness and rigidity. Some studies have shown that these properties can be evaluated by measuring the fluorescence spectra of the molecules. In fact, molecular rigidity is related to an increase in the fluorescence intensity (Albani, 2004), and molecular compactness is reflected in the diversity of peak distribution that tends to decrease and move to longer wavelengths due to molecular crosslinking (Albani, 2004). The two parameters can be evaluated using synchronous fluorescence (Peuravuori et al., 2002; Albani, 2004).

**Figure 4:**
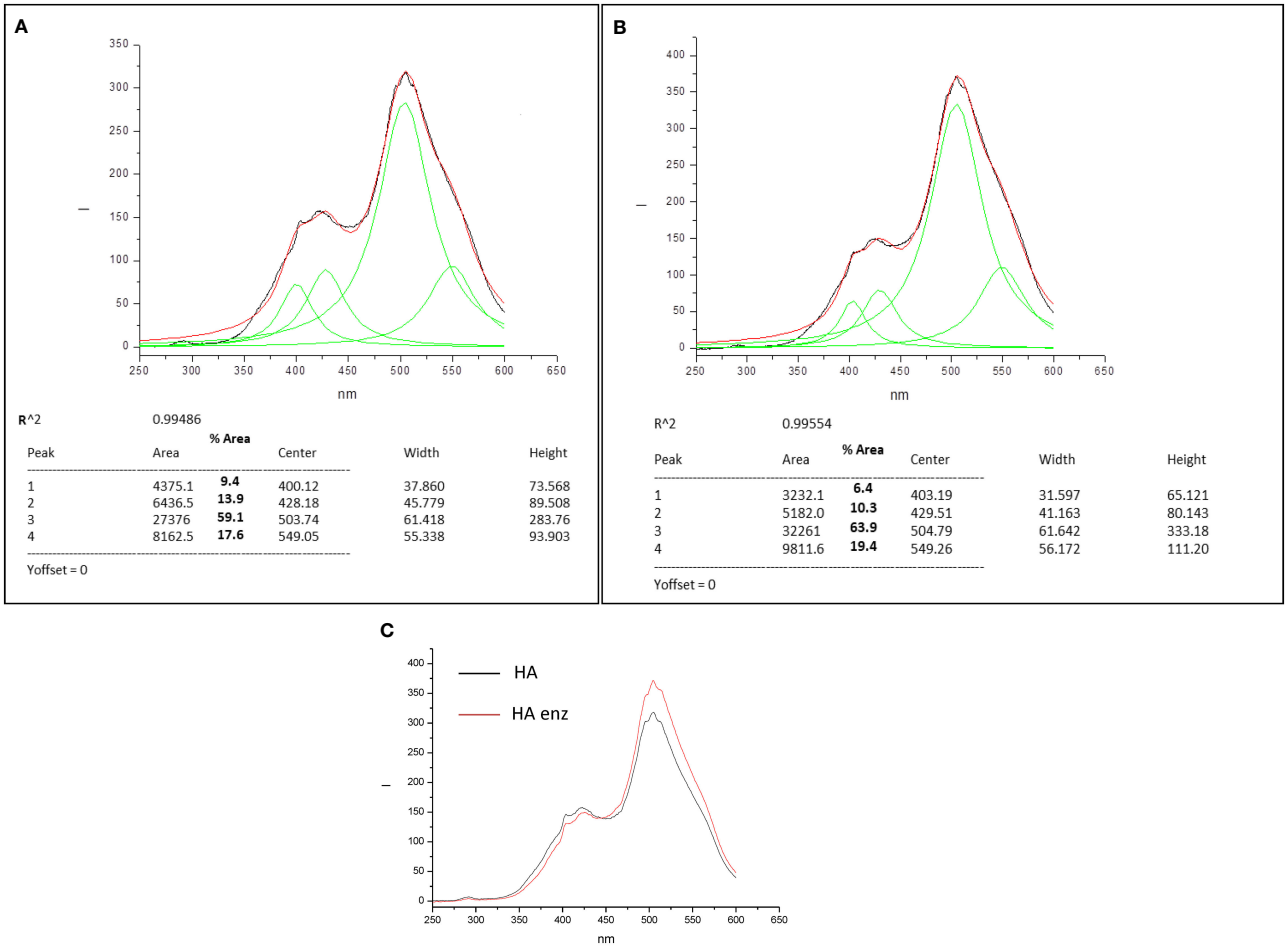
Synchronous Fluorescence spectra and convoluted peaks for HA **(A)** and HA enz **(B)**, and overlay spectrum HA vs HA enz **(C)**.

The synchronous fluorescence spectra of HA and HA enz presented four significant peaks, two around 400-430 nm and two around 500-550 nm (**Figures 4A, B**). Peuravuori et al. (2002) proposed that this range of peaks may correspond to polycyclic aromatic moieties with different degrees of conjugation; the longer the wavelength, the larger the conjugation degree. In this framework, the results indicate that laccase treatment led to more complex structural conformations, which is reflected in the increase in the relative percentage of the area for those associated with longer wavelength (503-504 nm and 549 nm) for HA enz (**Figures 4A, B**). The increase in molecular complexity is likely related to the laccase-induced molecular crosslinking that favors the formation of tighter conformations with fewer conformational degrees of freedom and higher potential rigid conjugation systems (Piccolo et al., 2005). Besides that, it is noteworthy that laccase treatment is also associated with an increase in fluorescence intensity that might be caused by an increase in molecular rigidity (Albani, 2004) (**Figure 4C**).

**Figure 5:**
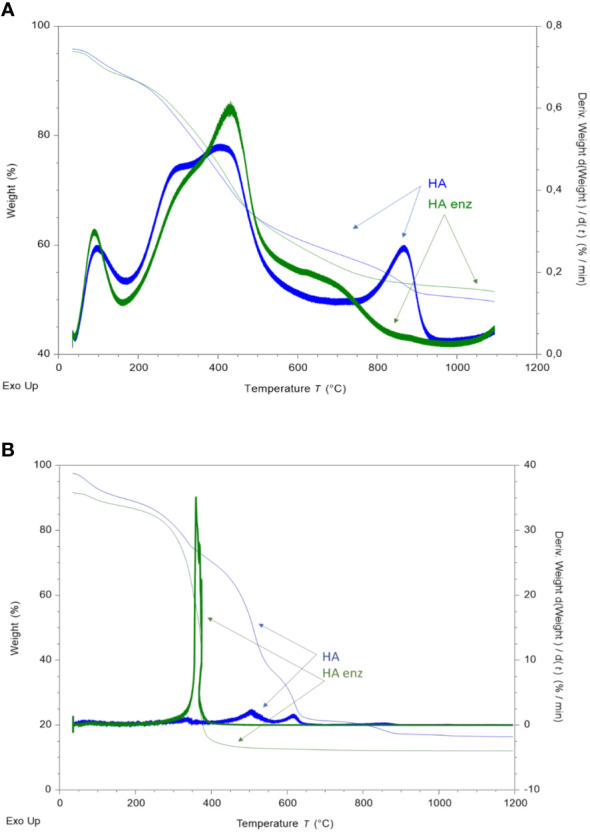
TG and DTG curves of the thermal decomposition of HA and HA enz under inert atmosphere **(A)**; and under oxidant atmosphere **(B)**.

The increase in conformational stability, molecular compactness, and rigidity is also reflected in the results obtained from TGA study. Under a non-oxidant atmosphere, it is observed a first peak around 160°C that corresponds to adsorbed-water evaporation and the volatilization of volatile compounds for HA and HA enz (**Figure 5A**). A doublet- peak is observed for HA and HA enz in the 200-450°C range. The first peak corresponds to the elimination of carboxylic, alcohol, methyl, and methylene groups and carbohydrate decomposition (Dell’Abate et al., 2003; Rotaru et al., 2008; Santos et al., 2018). It appears at 288°C for HA and 303°C for HA enz (**Figure 5A**). The second peak, around 400-600°C, corresponds to the poly-condensation and decomposition of aromatic moieties and volatile new-formed residues (Dell’Abate et al., 2003; Rotaru et al., 2008; Santos et al., 2018). Whereas HA presented an inflection point at 412°C, HA enz has this inflection point at higher temperatures (432°C) (**Figure 5A**). These results indicate that HA enz has higher thermal stability, likely related to the higher conformational stability and molecular compactness observed in the previously obtained results using other analytical techniques. In line with that, HA enz presented lesser mass losses in the 200-300°C range (10.42% of total losses) and higher mass losses in the range of 400-600°C (20.43%) than HA (12.36 and 17.63%, respectively).

However, HA and HA enz presented a different behavior under an oxidant atmosphere from that shown under an inert atmosphere (**Figure 5B**). Whereas HA enz presented a single peak in the range of temperatures before 400°C (250-400°C with the maximum at 378.17°C) that involves the loss of nearly 80% of the mass, HA presented two prominent peaks at higher temperatures of decomposition, within the range of 450 and 610°C (**Figure 5B**). These results indicate that HA enz is much more prone to be oxidized than HA. This fact might be related to the presence of more carboxylic groups in HA enz as a result of laccase treatment. Another parameter that can influence the rapid oxidation of HA enz is the specific surface of the molecule (Molto et al., 2006), which might be lower in the case of HA enz due to laccase-induced molecular crosslinking.

In consequence, these results indicate that laccase treatment modified the structural features of HA, increasing molecular compactness and rigidity (less conformational degrees of freedom) as well as the degree of hydrophobicity. These changes are also associated with an increase in carboxylic groups and a decrease in phenolic groups. All these changes are consistent with a decrease in the non-covalent intermolecular interactions present in HA-molecular assembly and an increase in intermolecular covalent interactions and the macromolecular character resulting from laccase treatment of HA.

### 3.2 Laccase treatment affected the ability of HA to promote plant growth but not Cu-complexing ability

**Figure 6:**
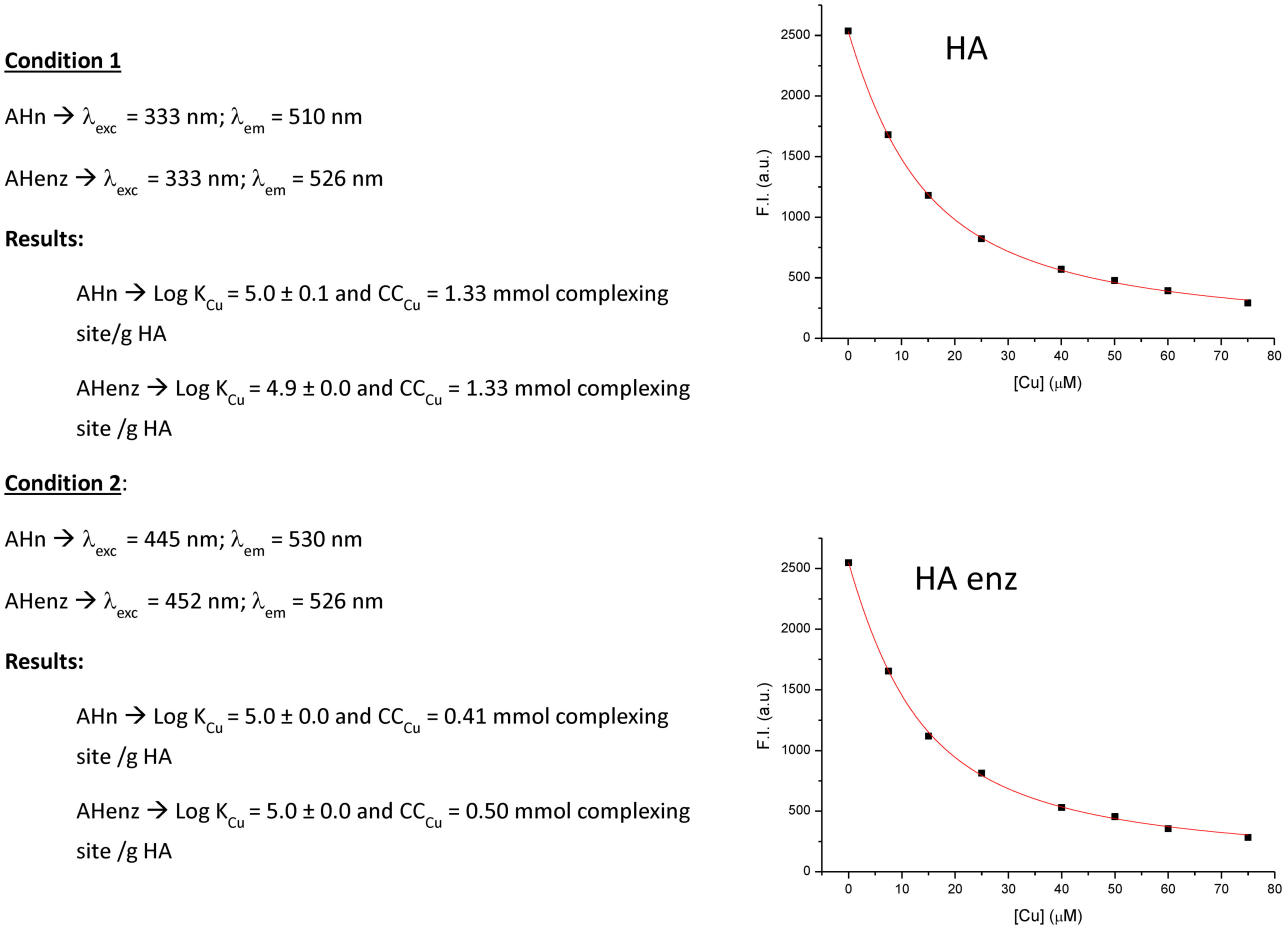
Study on the complexation of Cu by HA and HA enz. Stability constants and complexation ability expressed as the concentration of complexing sites per g of humic sample.

We have used Cu complexation to evaluate possible changes in HA chemical activity caused by laccase treatment. The results showed that the oxidative polymerization induced by laccase was not associated with changes in the Cu complexation ability of HA since any differences between HA and HA enz were observed (**Figure 6**). The value of stability constants (log K) was around 5, in line with the results of other studies employing different HA that obtained a value within the 3-5 range (Plaza et al., 2005; Garcia-Mina, 2006; Fuentes et al., 2013). Although significant, this result indicates that the changes in the relative proportion of carboxylic and phenol groups induced by laccase treatment did not have relevant consequences on Cu complexation capacity. This finding also suggests that the particular conformation of HA in solution does not have a relevant incidence on HA metal complexation. This fact might be due to the presence of the complexing sites on HA surface (Olaetxea et al., 2021).

Regarding HA biological activity evaluated by its ability to promote plant growth, our working hypothesis is that the singular conformational features of HA, mainly its supramolecular-like conformation, and the events triggered by the HA – root interaction (conformational changes that may include molecular disaggregation) are directly related to its ability to modify plant growth. As described above, the treatment of HA with laccase produced a modified HA enz with higher hydrophobicity, compactness and rigidity, and less conformational freedom. In this framework, comparing the effects on plant growth of HA and HA enz might allow us to evaluate the importance of molecular aggregation in HA in its ability to affect plant growth. To investigate this hypothesis, we have studied the effects of HA and HA enz on the growth of two plant species: cucumber and Arabidopsis.

**Figure 7:**
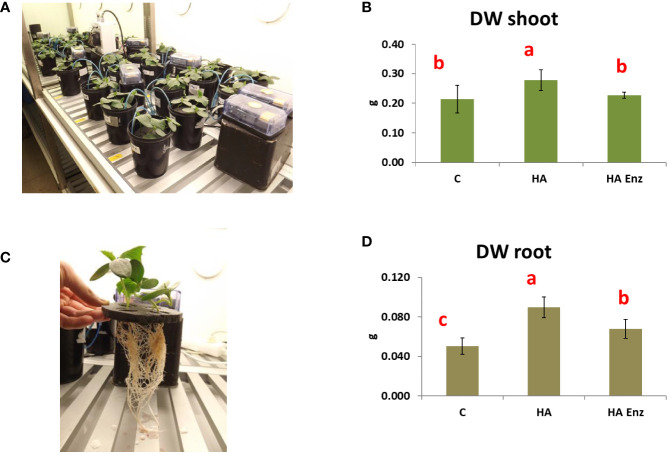
Effect of HA and HA enz root application on the shoot dry weight (DW) (mean ± SD) **(A, B)**, and root dry weight (DW) (mean ± SD) **(C, D)** of cucumber plants. Pictures correspond to one representative replicate. (Different letters correspond to significant differences for p≤ 0.05. LSD Fisher test. Five replicates of one plant per replicate).

**Figure 8:**
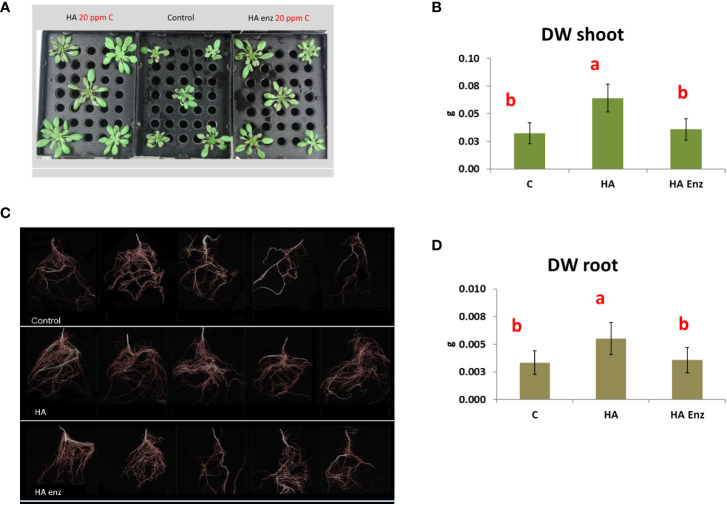
Effect of HA and HA enz root application on the shoot dry weight (DW) (mean ± SD) **(A, B)**, and root dry weight (DW) (mean ± SD) **(C, D)**, of Arabidopsis plants. Pictures (WinRHIZO image system) correspond to one representative replicate. (Different letters correspond to significant differences for p≤ 0.05. LSD Fisher test. Five replicates of one plant per replicate).

**Figure 9:**
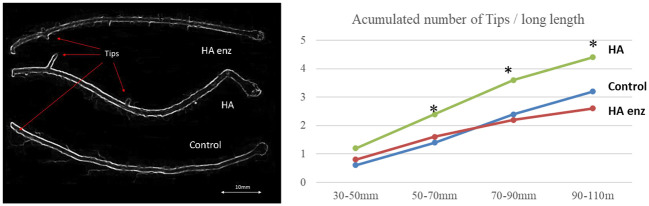
Effects of HA and HA enz on lateral root formation (mean). (* significant differences for p≤ 0.01. LSD Fisher test. Five replicates with one plant per replicate).

In good agreement with previous studies (Mora et al., 2010; Olaetxea et al., 2015; Olaetxea et al., 2018), the results showed that HA caused an increase in both shoot dry weight and root dry weight in the two plant species (**Figures 7**, **8**). However, the results indicated that the structural modifications introduced in HA structural features by Laccase treatment avoided the HA capacity to increase shoot and root growth in both plant species (**Figures 7**, **8**). Likewise, HA enz did not present the capacity to promote lateral root proliferation (**Figure 9**). These results indicate that either molecular aggregation in HA or the concentration of phenol/carboxylic groups are directly involved in the HA capacity to stimulate plant growth. Considering that the changes in phenol and carboxylic groups were partial (carboxylic and phenol groups are still present after laccase treatment), while the reduction in biological activity was complete, the results suggest that the higher conformational freedom of HA associated with its weakly bond stabilized aggregates might have an essential role in the HA capacity to stimulate plant growth. In this framework, this fact could be related to changes in the molecular conformational configuration of HA that may include the induction of HA molecular disaggregation, with the two processes resulting from the interaction of HA with root exudates in the rhizosphere. In order to investigate this fact, we have studied the molecular size and conformational features of HA and HA enz samples upon their interaction with cucumber roots, using DLS and synchronous fluorescence, respectively. In this framework, while HA can undergo molecular disaggregation (Baigorri et al., 2007), laccase-treated HA, enz HA, do not (Piccolo et al., 2005).

### 3.3 The interaction with plant roots did not cause molecular disaggregation of HA and HA enz, but modified their molecular conformation in solution

**Figure 10:**
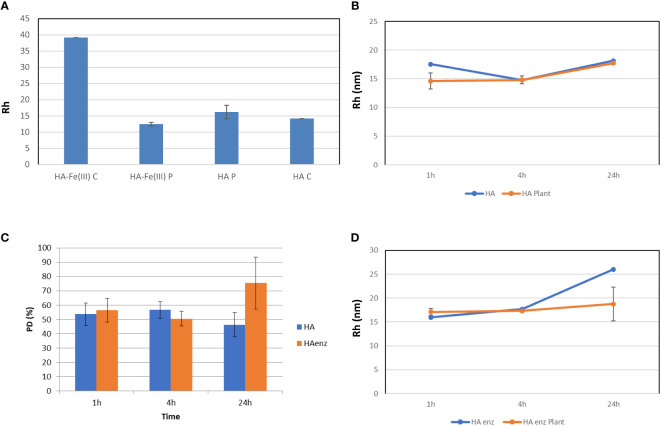
Size distribution analysis by DLS. **(A)** Changes in the Rh (nm) value of HA-Fe(III) after 1 h of contact with cucumber plant roots compared with that of HA in samples in nutrient solution without plant (HA-Fe(III) C and HA C), and with plant (HA-Fe(III) P and HA P); **(B)** Rh values (nm) of HA in nutrient solution with plant (HA Plant) and without plant (HA) over time; **(C)** Polydispersity index for HA and HA enz in nutrient solution in the presence of plant; **(D)** Rh (nm) values of HA enz in nutrient solution with plant (HA enz Plant) and without plant (HA enz) over time. (mean ± SD. Five replicates per treatment). Samples filtered through 0.1 µm before Rh determination.

In a first study, we checked the ability of DLS to detect molecular size changes of HA in nutrient solution, in the presence and absence of plant roots. The interest in employing DLS for the study of HA size distribution in nutrient solution arises from the limitations of HPSEC to measure HA size under these experimental conditions due to the dilution of the sample. Our experience indicated that obtaining consistent and reproducible HPSEC spectra requires a concentration of organic carbon around 800 mg L^-1^ (injection volume of 100 µl) (Fuentes et al., 2006). However, the organic carbon concentration in HA samples in the nutrient solution is around 80 mg L^-1^. To test DLS performance, we studied the size changes of a Fe (III) – HA complex after its interaction with cucumber plant roots and the root uptake of HA complexed-Fe. The uptake of Fe from Fe-HA was assessed by the concentration of soluble Fe in plant shoots that was similar to that of the plants treated with Fe-EDDHA (**Figure S2**). The results showed that the root uptake of HA complexed-Fe and the associated Fe-HA hydrolysis, releasing free HA, was linked to a significant decrease of the Rh value of complexing HA that became similar to that of HA alone after one hour from the onset of treatments (**Figure 10A**). These results showed that DLS is a good technique for monitoring molecular size changes in the nutrient solution in the presence of plant roots under our experimental conditions.

**Figure 11:**
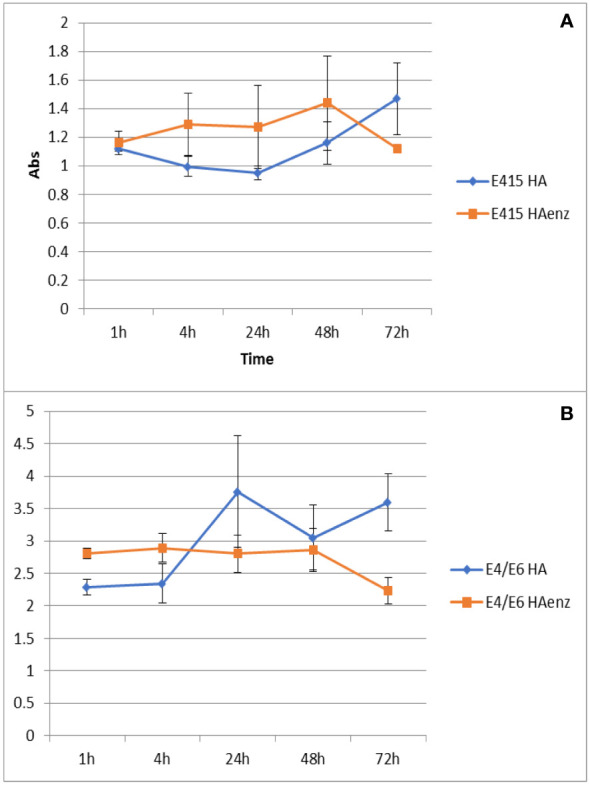
Variation of Abs at 415 nm **(A)**, and E4/E6 ratio **(B)** over time. (mean ± SD. Five replicates per treatment).

**Figure 12:**
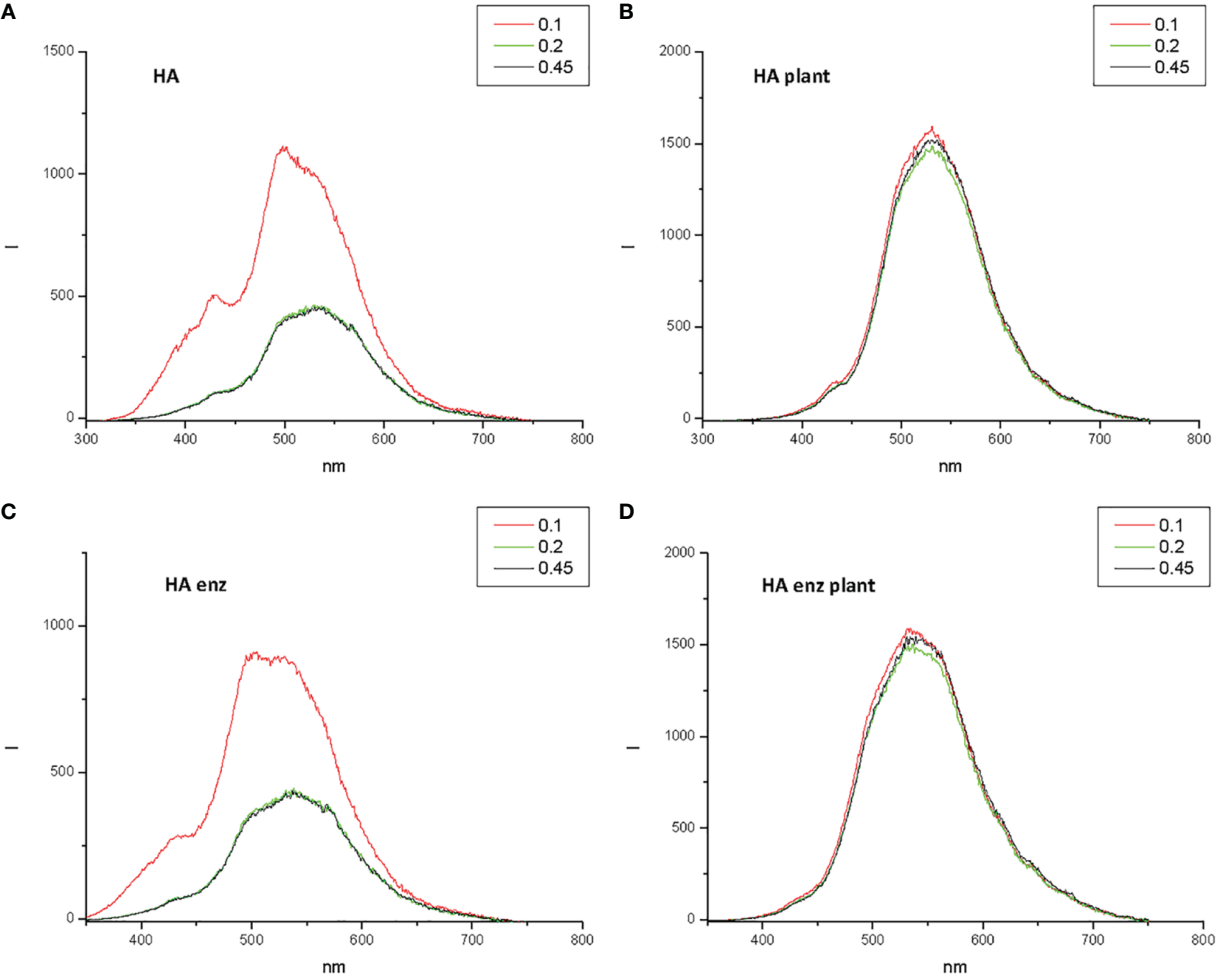
Synchronous Fluorescence spectra for HA **(A)** and HA enz **(C)** in nutrient solution without plant, and for HA **(B)** and HA enz **(D)** in nutrient solution with plant. The spectrum in red corresponds to the sample filtered through 0.1 µm. The spectrum in green corresponds to the sample filtered through 0.2 µm. The spectrum in black corresponds to the sample filtered through 0.45 µm.

Next, the experiments to evaluate the effect of the interaction of HA and HA enz with plant roots on the size distribution of HA and HA enz were carried out in cucumber. Unexpectedly, we did not observe significant changes in the average molecular size of either HA or HA enz, monitored as the value of Rh in solution, upon root interaction (**Figures 10B, C**; **Figure S3**). In all cases, the index of polydispersity was, in average, 50% (**Figure 10C**). The possible influence of HA absorption by roots was evaluated by the molecular absorption at 415 nm of the nutrient solution. The results did not show significant changes in E 415 (**Figure 11A**), thus indicating no significant absorption of HA by roots. However, regarding E4/E6 ratio, whereas HA enz did not show noticeable changes, HA presented an increase in the ratio over time (**Figure 11B**). This result is in line with that other authors obtained on the interaction of HS with polycarboxylic acids mimicking root exudates (Piccolo et al., 1996; Canellas et al., 2008; Olaetxea et al., 2018). These changes were interpreted as resulting from the molecular disaggregation of HS (Piccolo et al., 1996; Canellas et al., 2008; Olaetxea et al., 2018). However, the relationship between molecular weight and E4/E6 ratio is unclear (Fuentes et al., 2006). It is possible that the changes in the E4/E6 ratio resulted from the conformational changes experienced by HA upon its interaction with plant roots. Although the DLS study indicates that neither HA nor HA enz experience molecular disaggregation upon interacting with plant roots, E4/E6 values show apparent conformation changes in HA induced by its interaction with plant roots (**Figures 10**, **11**). The SF study supports this conclusion. Thus, the interaction with plant roots and probably with root exudates caused significant changes in SF spectra that can be summarized in the appearance of a central peak with a maximum at 550 nm, which presented higher fluorescence intensity than the peaks of HA and HA enz in nutrient solution but without plant (**Figures 12A–D**). It is noteworthy that these changes observed for HA and HA enz are very similar to each other (**Figures 12A–D**). In principle, the increase in fluorescence intensity might be related to an increase in molecular rigidity that may result from the molecular crosslinking of all HA fractions to form a less heterogeneous molecular system (Piccolo et al., 2005). This process might be related to the presence in root exudates of oxidative enzymes similar to laccase or other oxidants (Vives-Peris et al., 2019). This fact involves the transformation of the supramolecular-like structural conformation of HA into new conformations with high macromolecular character in the root area of the rhizosphere and the rhizosheath. These results are in line with other studies showing the presence of either supramolecules or macromolecules in SH systems in solution (Baigorri et al., 2007). This process can have relevant ecological meaning since it suggests that the HS closer to the root (rhizosphere and rhizosphere-rhizosheath) are modified at the root surface, probably by root exudates and microorganisms, and their biological activity might change due this process. In consequence, the HA included in the rhizosphere (rhizosphere-rhizosheath) soil and those present in non-rhizosphere (and non-rhizosphere-rhizosheath) soil probably present differences concerning their structural and biological features. This conclusion is in line with studies showing that the composition of DOM in rhizosheath soil differs from that of non-rhizosheath soil (Mo et al., 2022) and that DOM transformation is closely linked to root and microorganism’s activities (Hu et al., 2022).

In summary, the SF study showed that the interaction of HA and HA enz with plant roots is not associated with molecular disaggregation but with significant changes in the molecular configuration of the two humic samples. The new conformational configuration resulting from HA – root interaction indicates the formation of more complex and homogeneous structures with higher molecular rigidity. These changes are experienced by HA and HA enz. However, E4/E6 values indicate that HA, but not HA enz, experienced significant conformational changes associated with root interaction. The results suggest that these conformational changes might be essential for the HA ability to promote plant development. The results also indicate that in natural environments we probably have two main classes of HS: those interacting with plant roots that seem to have a more pronounced macromolecular character, and those that do not interact with plant roots that present a more pronounced molecular aggregation (molecular assemblies).

It also becomes clear that the structural modifications induced by laccase treatment led to the loss of biological activity of HA. As indicated above, laccase treatment caused a significant increase in molecular compactness, rigidity, stability, and hydrophobicity. These changes were also linked to concomitant changes in the relative proportion of carboxylic and phenolic groups. In principle, all these structural changes are associated with intra- and inter-molecular phenol-mediated crosslinking accompanying laccase treatment. In this framework, the different conformational configuration of HA (high molecular aggregation and supramolecular-like character) and HA enz (high macromolecular character) in solution probably plays a relevant role in the ability of HA to promote the biochemical events in plant roots responsible for the whole action on plant growth. However, a possible role of the relative proportion of carboxylic and phenolic groups in HA-mediated promoting action of plant growth cannot be ruled out.

## Data availability statement

The original contributions presented in the study are included in the article/**Supplementary Material**. Further inquiries can be directed to the corresponding author.

## Author contributions

JA, DH, general experimentation and data treatment and discussion. MO, RB, OU, MG, JE, MM, AZ, plant experiments, physico-chemical characterization, and data discussion. MF, metal complexation experiments. EB, microscopy, GGA, DLS study. JIA, thermal and wettability analyses. JG-M, conceptual design, data interpretation, and discussion. All authors contributed to the article and approved the submitted version.
