# Supplementary material for: The molecular conformation, but not disaggregation, of humic acid in water solution plays a crucial role in promoting plant development in the natural environment
Source: Front Plant Sci. 2023 May 3;14:1180688. doi: 10.3389/fpls.2023.1180688 (PMC10190593; doi:10.3389/fpls.2023.1180688)
Supplement: Supplementary file 1 [file DataSheet_1.docx]

**Supplementary Data**

Figure S1. Size distributions obtained by DLS for: A) HA, and B) HA enz as a function of the pH (fraction filtered by 0.1 um in both cases).

Figure S2. Concentration of soluble Fe in the leaves of treated plants. (mean ± SD. Five replicates per treatment). Treatments with HA1 (HA purified with fluorhydric acid treatment), HA, HA enz and NS (nutrient solution) have Fe as Fe-EDDHA.


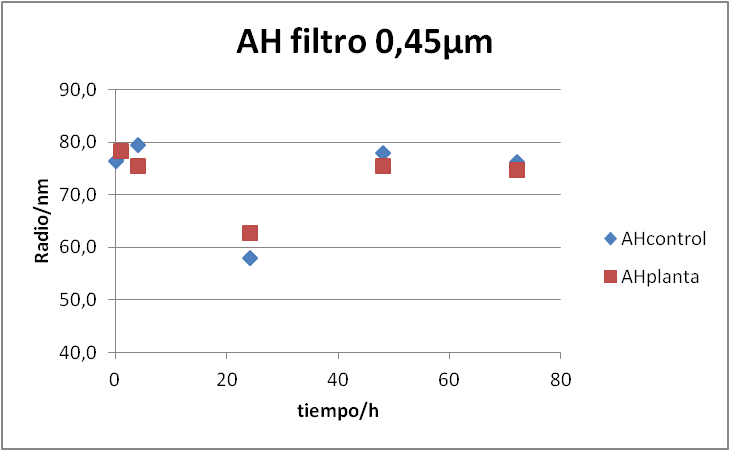


**HA**

**HA plant**

**Time (h)**

**Rh (nm)**


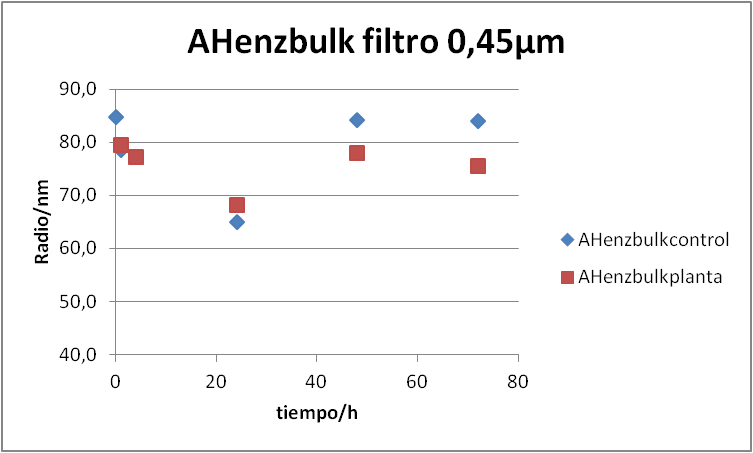


**HA enz**

**HA enz plant**

**Time (h)**

**Rh (nm)**

Figure S3. Variation of Rh (nm) of HA and HA enz in the nutrient solution with and without plant. Mean of five replicates per treatment. Samples were filtered through 0.45 µm before DLS analysis.
